# Evaluating multiple sclerosis severity loci 30 years after a clinically isolated syndrome

**DOI:** 10.1093/braincomms/fcae443

**Published:** 2024-12-05

**Authors:** Nitin Sahi, Lukas Haider, Karen Chung, Ferran Prados Carrasco, Baris Kanber, Rebecca Samson, Alan J Thompson, S Anand Trip, Wallace Brownlee, Olga Ciccarelli, Frederik Barkhof, Carmen Tur, Henry Houlden, Declan Chard

**Affiliations:** NMR Research Unit, Queen Square Multiple Sclerosis Centre, Department of Neuroinflammation, University College London Queen Square Institute of Neurology, London WC1N 3BG, UK; NMR Research Unit, Queen Square Multiple Sclerosis Centre, Department of Neuroinflammation, University College London Queen Square Institute of Neurology, London WC1N 3BG, UK; Department of Biomedical Imaging and Image Guided Therapy, Medical University Vienna, 1090 Vienna, Austria; NMR Research Unit, Queen Square Multiple Sclerosis Centre, Department of Neuroinflammation, University College London Queen Square Institute of Neurology, London WC1N 3BG, UK; NMR Research Unit, Queen Square Multiple Sclerosis Centre, Department of Neuroinflammation, University College London Queen Square Institute of Neurology, London WC1N 3BG, UK; Centre for Medical Image Computing (CMIC), Department of Medical Physics and Biomedical Engineering, University College London, London WC1E 6BT, UK; E-Health Center, Universitat Oberta de Catalunya, Barcelona 08018, Spain; NMR Research Unit, Queen Square Multiple Sclerosis Centre, Department of Neuroinflammation, University College London Queen Square Institute of Neurology, London WC1N 3BG, UK; Centre for Medical Image Computing (CMIC), Department of Medical Physics and Biomedical Engineering, University College London, London WC1E 6BT, UK; Department of Clinical and Experimental Epilepsy, University College London, London WC1N 3BG, UK; NMR Research Unit, Queen Square Multiple Sclerosis Centre, Department of Neuroinflammation, University College London Queen Square Institute of Neurology, London WC1N 3BG, UK; NMR Research Unit, Queen Square Multiple Sclerosis Centre, Department of Neuroinflammation, University College London Queen Square Institute of Neurology, London WC1N 3BG, UK; NMR Research Unit, Queen Square Multiple Sclerosis Centre, Department of Neuroinflammation, University College London Queen Square Institute of Neurology, London WC1N 3BG, UK; NMR Research Unit, Queen Square Multiple Sclerosis Centre, Department of Neuroinflammation, University College London Queen Square Institute of Neurology, London WC1N 3BG, UK; National Institute for Health and Care Research (NIHR), University College London Hospitals (UCLH) Biomedical Research Centre, London W1T 7DN, UK; NMR Research Unit, Queen Square Multiple Sclerosis Centre, Department of Neuroinflammation, University College London Queen Square Institute of Neurology, London WC1N 3BG, UK; National Institute for Health and Care Research (NIHR), University College London Hospitals (UCLH) Biomedical Research Centre, London W1T 7DN, UK; NMR Research Unit, Queen Square Multiple Sclerosis Centre, Department of Neuroinflammation, University College London Queen Square Institute of Neurology, London WC1N 3BG, UK; Centre for Medical Image Computing (CMIC), Department of Medical Physics and Biomedical Engineering, University College London, London WC1E 6BT, UK; National Institute for Health and Care Research (NIHR), University College London Hospitals (UCLH) Biomedical Research Centre, London W1T 7DN, UK; Department of Radiology and Nuclear Medicine, VU University Medical Centre, Amsterdam 1081 HV, The Netherlands; NMR Research Unit, Queen Square Multiple Sclerosis Centre, Department of Neuroinflammation, University College London Queen Square Institute of Neurology, London WC1N 3BG, UK; MS Centre of Catalonia (Cemcat), Vall d’Hebron Institute of Research, Vall d’Hebron Barcelona Hospital Campus, Barcelona 08035, Spain; Department of Neuromuscular Diseases, UCL Queen Square Institute of Neurology, Queen’s Square House, London WC1N 3BG, UK; NMR Research Unit, Queen Square Multiple Sclerosis Centre, Department of Neuroinflammation, University College London Queen Square Institute of Neurology, London WC1N 3BG, UK; National Institute for Health and Care Research (NIHR), University College London Hospitals (UCLH) Biomedical Research Centre, London W1T 7DN, UK

**Keywords:** multiple sclerosis, disease progression, severity, genetics, phenotype

## Abstract

The first genome-wide significant multiple sclerosis severity locus, rs10191329, has been pathologically linked to cortical lesion load and brain atrophy. However, observational cohorts such as MSBase have not replicated associations with disability outcomes, instead finding other loci. We evaluated rs10191329 and MSBase loci in a unique cohort of 53 people followed for 30 years after a clinically isolated syndrome, with deep clinical phenotyping and MRI measures of inflammation and neurodegeneration. After 30 years, 26 had developed relapsing-remitting multiple sclerosis, 15 secondary progressive multiple sclerosis and 12 remained diagnosed with a clinically isolated syndrome. Genetic associations with disease severity (age-related multiple sclerosis severity score and Expanded Disability Status Scale), disease course and brain MRI features (white matter lesions, cortical lesions and grey matter fraction) were investigated using regression models and survival analyses. rs10191329 was not associated with multiple sclerosis severity, secondary progressive multiple sclerosis diagnosis or brain MRI features at 30 years. Similarly, MSBase loci were not associated with 30-year disease severity, although rs73091975 was significantly associated with lower 14-year age-related multiple sclerosis severity score in those developing multiple sclerosis. Given that effect sizes for both rs10191329 and rs73091975 were greatest between 14 and 20 years, these findings suggest genetic effects on multiple sclerosis severity may interact non-linearly with disease duration.

## Teaser

Sahi *et al*. evaluated rs10191329 and MSBase genetic variants with multiple sclerosis severity 30 years after the first symptoms. No loci were associated with 30-year disability, MRI outcomes or disease course, although rs73091975 was associated with age-related multiple sclerosis severity at 14 years, suggesting potential for non-linear genetic effects over time.

## Graphical Abstract

**Graphical Abstract:**
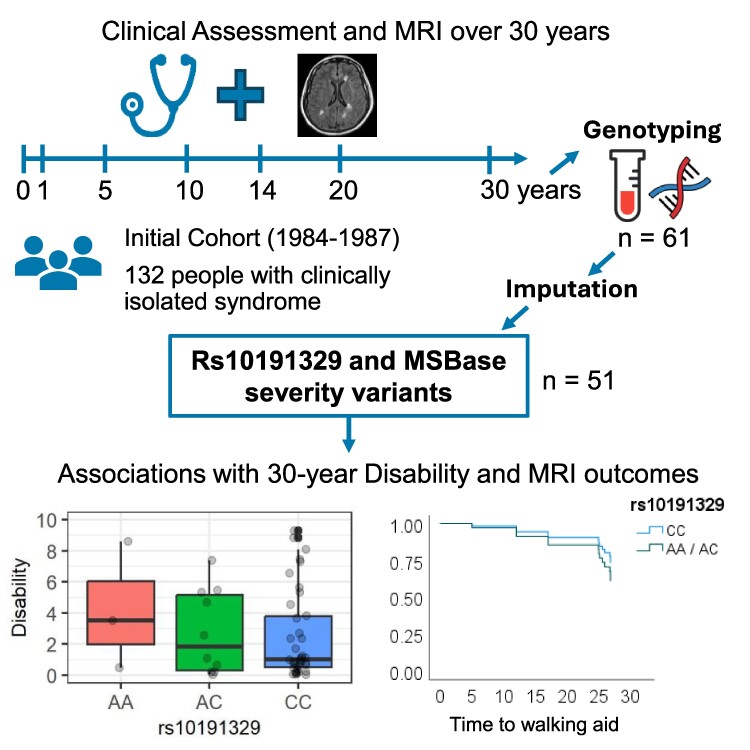

## Introduction

Multiple sclerosis is an inflammatory and neurodegenerative CNS disorder with heterogeneous pathological features and clinical course. Genetics contribute to the variability of multiple sclerosis severity, but its role is not straightforward to resolve as effects may be mediated by different elements of multiple sclerosis pathology.

The International Multiple Sclerosis Genetics Consortium (IMSGC) recently identified rs10191329 (near *DYSF-ZNF68*) as the first variant associated with age-related multiple sclerosis severity (ARMSS) at genome-wide significance (*P* < 5 × 10^−8^) in a discovery cohort of 12 584 people with multiple sclerosis (mean disease duration of 18.2 years) and replicated in 9805 cases (mean disease duration of 15.8 years).^1^ rs10191329^A^ carriage was associated with faster Expanded Disability Status Scale (EDSS) worsening, and homozygosity was associated with shorter time to requiring a walking aid (EDSS 6.0), as well as higher cortical lesion and brainstem lesion counts in independent autopsy samples from 290 multiple sclerosis cases.^1^ Subsequent studies looked to determine clinically useful signatures, while some also attempted to replicate disability associations. In 748 patients with clinically isolated syndrome (CIS) or relapsing-remitting multiple sclerosis (RRMS) with mild disability (median EDSS 1.0) rs10191329 was associated with 28% higher yearly change in percentage brain volume per rs10191329^A^ allele over a median inter-scan interval of 3 years.^2^ The authors observed no association with EDSS or change in EDSS (disease duration not specified), despite significant overlap of their cohort with the IMGSC study (32% overlap with discovery cohort and 87% overlap with replication cohort). Similarly, there was no association between rs10191329 and longitudinal disease severity in 1813 relapse-onset multiple sclerosis patients from the MSBase register (median disease duration of 18.1 years and median follow-up of 11.7 years),^3^ which yielded suggestive (*P* < 5 × 10^−5^) but non-overlapping variants compared with the IMSGC study.^4^ Kreft *et al*.^5^ also did not detect an association between rs10191329 and ARMSS or time to EDSS milestones in 1455 South Wales multiple sclerosis registry patients (mean disease duration of 14 years), but did report 2 MSBase variants (rs7289446^G^ and rs868824^C^) associated with disability scores. Although these large-scale cohorts have mean disease durations over a decade, it can take two or more for the ultimate clinical outcome of a person with multiple sclerosis to be clear,^6^ making it difficult to unpick the contributions of neuroinflammation and neurodegeneration, which may dominate at different stages of the disease, and both impact on clinical outcomes.

We previously reported on MRI and clinical outcomes in a unique cohort of people followed prospectively for 30 years after a CIS,^7^ finding clear differences in the accrual of brain lesions, brain atrophy and disability progression by clinical course.^8^ Notably, the presence of infratentorial lesions within the first year of disease was the strongest early predictor of developing secondary progressive multiple sclerosis (SPMS),^7^ while at 30 years, cortical lesions best explained long-term disability as reflected by EDSS.^9^ A subset of this cohort underwent genetic testing at 30-year follow-up with two genetic variants rs4866550 (*IRX1)* and rs4803766 *(PVRL2/NECTIN2)* associated with long-term disability worsening, cortical lesions and brain atrophy but only tested fixed markers within the genotyping array.^10^ Following genotype imputation, we examined rs10191329 and MSBase severity variants in this cohort. The aims were to replicate associations with 30-year outcomes of disease severity, pathological and MRI findings (cortical lesions and brain atrophy) and investigate if any variant could predict long-term disease course in a homogenous cohort of people followed from the time of their CIS.

## Materials and methods

### Study cohort

One hundred thirty-two people were recruited following a CIS at the National Hospital of Neurology and Neurosurgery and Moorfields Eye Hospital in the 1980s and underwent clinical assessment and MRI brain at baseline, 1, 5, 10, 14, 20 and 30 years, as previously described.^7-9^ At 30-year follow-up, 61 participants gave blood samples for genotyping.^7,10^ Following genotype imputation (see below), 53 participants were included in this study; 41 diagnosed with multiple sclerosis and 12 remaining as CIS according to 2010 McDonald diagnostic criteria^11^ (Table 1). EDSS was assessed at baseline, 5-, 10-, 14-, 20- and 30-year follow-ups by clinical examination (or telephone at later timepoints) and determined retrospectively if missing at a given follow-up.^7^ EDSS scores were used to calculate corresponding ARMSS at each timepoint from global ARMSS matrix.^12^

**Table 1:** Clinical and radiological characteristics by 30-year clinical diagnosis

		All genotyped participants	Participants post-imputation	Diagnosis at 30 years		
				CIS	RRMS	SPMS
Number		61	53	12	26	15
Age (years)		60.9 ± 6.5	61.0 ± 6.7	60.6 ± 6.8	60.6 ± 6.6	61.9 ± 6.7
Female		41 (67%)	36 (68%)	7 (58%)	18 (69%)	11 (73%)
Age at onset (years)		30.2 ± 6.4	30.2 ± 6.6	29.5 ± 7.5	29.9 ± 6.6	31.6 ± 6.2
Disease duration (years)		30.8 ± 0.9	30.9 ± 0.9	30.8 ± 0.9	31.0 ± 0.9	30.8 ± 0.9
CIS type	Optic Neuritis	31 (51%)	26 (49%)	7 (58%)	11 (42%)	8 (53%)
	Spinal cord	21 (34%)	18 (34%)	4 (33%)	9 (35%)	5 (33%)
	Brainstem	9 (15%)	9 (17%)	1 (8%)	6 (23%)	2 (13%)
Baseline EDSS^a^	Mean ± SD	2.6 ± 1.3	2.6 ± 1.3	3.3 ± 1.2	2.3 ± 1.0	2.5 ± 1.7
	Median (IQR)	3.0 (2.0–3.125)	3.0 (2.0–3.5)	3.0 (3.0–3.5)	2.0 (2.0–3.0)	3.0 (1.0–3.5)
Time CIS to RRMS (years)		5.8 ± 6.0	5.7 ± 5.8	NA	6.7 ± 6.7	3.8 ± 2.9
Time CIS to SPMS (years)		19.6 ± 5.5	19.6 ± 5.5	NA	NA	19.6 ± 5.5
EDSS at 30 years	Mean ± SD	2.7 ± 2.4	2.9 ± 2.5	1.1 ± 1.1	1.8 ± 1.5	6.2 ± 0.8
	Median (IQR)	2.0 (1.0–5.5)	2.0 (1.0–5.75)	0.75 (0.0–2.0)	1.5 (1.0–2.0)	6.0 (6.0–6.5)
DMT usage	Yes	9	7	0	2	5
	No	52	46	12	24	10
Baseline WM lesion volume (ml)		1.17 ± 2.37	1.31 ± 2.54	0.13 ± 0.20	0.86 ± 0.81	2.46 ± 3.94
WM lesion volume at 30 years (ml)		16.49 ± 14.23	17.55 ± 14.25	5.86 ± 9.41	17.53 ± 11.81	26.95 ± 14.64
Cortical lesions at 30 years (*n*)		0.7 ± 1.3	0.7 ± 1.3	0.0 ± 0.0	0.0 ± 0.2	2.2 ± 1.5
GMF at 30 years (%)		43.4 ± 1.3	43.4 ± 1.3	43.8 ± 1.2	43.7 ± 0.9	42.5 ± 1.7

Mean ± standard deviations unless stated otherwise.

IQR, interquartile range; WM, white matter; NA, not available.

^a^Baseline EDSS was recorded during initial CIS presentation.

This study was approved by the National Research Ethics Service (15/LO/0650). All participants gave written informed consent.

### MRI acquisition and analysis

MRI methods have been detailed previously.^7-10^ Briefly, 30-year follow-up was undertaken using a 3T MRI scanner (Philips Achieva) including 3D fluid-attenuated inversion recovery and T1-weighted volumetric images (both 1 × 1 × 1 mm^3^), T2-weighted axial scans (0.5 × 0.5 × 3 mm^3^) and phase-sensitive inversion recovery (PSIR) sequences (0.5 × 0.5 × 2 mm^3^; full acquisition parameters previously detailed).^9^ White matter lesion volume measurements were available from earlier studies.^9,13^ Cortical lesions were manually counted on PSIR blinded to clinical status,^9^ and grey matter fraction (GMF) was calculated using an atlas-based segmentation following T1-hypointense white matter lesion filling.^14,15^

### Genotyping and imputation

DNA was genotyped using the Illumina Infinium GSA-24 v3.0 Beadchip (San Diego, CA, USA) and assembled in GenomeStudio v2.0.5 (llumina) as previously outlined.^10^ Quality control procedures were performed in PLINK (version 1.90, Boston, MA, USA)^16^ excluding variants deviating from Hardy–Weinberg equilibrium (*P* < 10^−6^), minor allele frequency <0.01, missingness per variant >0.02, missingness per individual >0.045, excess heterozygosity (>3 SD from mean) and removal of duplicates.

SHAPEIT2 (version 2.r904, Oxford, UK)^17^ was used for phasing prior to imputation with IMPUTE2 (version 2.3.2, Oxford, UK)^18^ using the 1000 genomes Phase 3 reference panel. Overall, 488 021 variants and 53 people passed filters with a total genotyping rate of 0.999114. rs10191329 was imputed successfully in 51 individuals (genotyping rate 0.962264). Seven variants (rs7289446, rs1207401, rs7758683, rs56194930, rs73091975, rs295254 and rs11057374) associated with ARMSS from MSBase^4^ passed filters and post-imputation quality control (Supplementary Tables 1–3).

### Statistical analysis

Linear regression models were built with 30-year EDSS, ARMSS, cortical lesions or brain volume (GMF) as the dependent variable (one at a time) and rs10191329^A^ dosage (additive model) as the independent variable. Logistic regression models assessed the relationship between rs10191329 and binary outcome of ‘diagnosis of SPMS (“yes” or “no”) at 30 years’ as the dependent variable. MSBase variants were investigated with the relevant risk allele dosage replacing rs10191329^A^ as the independent variable. Box-Tidwell test was used to test linearity assumptions of continuous predictors (age) with their logit.

Exploratory analyses assessed associations of rs10191329^A^ dosage with disability measures at earlier timepoints (0, 5, 10, 14 and 20 years) using regression models and time to disability [EDSS 4.0 (maximum walking ability 500 m without aid) and EDSS 6.0 (requiring a walking aid)] using survival analysis with Cox proportional hazards models. Dominant genetic models were assumed to maximize statistical power for survival analyses due to the limited number of outcome events given the cohort size. Assumptions for Cox proportional hazards models were met (Supplementary Fig. 1).

Age, sex, disease-modifying therapy (DMT) use (‘yes’ or ‘no’) and smoking history (‘current’, ‘ex-smoker’ or ‘never’) were included as covariates in all models.^10^

SPSS (Version 27, Chicago, IL, USA) and RStudio (Version 1.1.463, Boston, MA, USA) were used for statistical analysis and data visualization. As a candidate gene study, statistical tests were two-tailed with *P*-values <0.05 considered statistically significant, without correction for multiple testing.

## Results

### No association of rs10191329 with multiple sclerosis disease severity or radiological outcomes at 30 years

**Figure 1:**
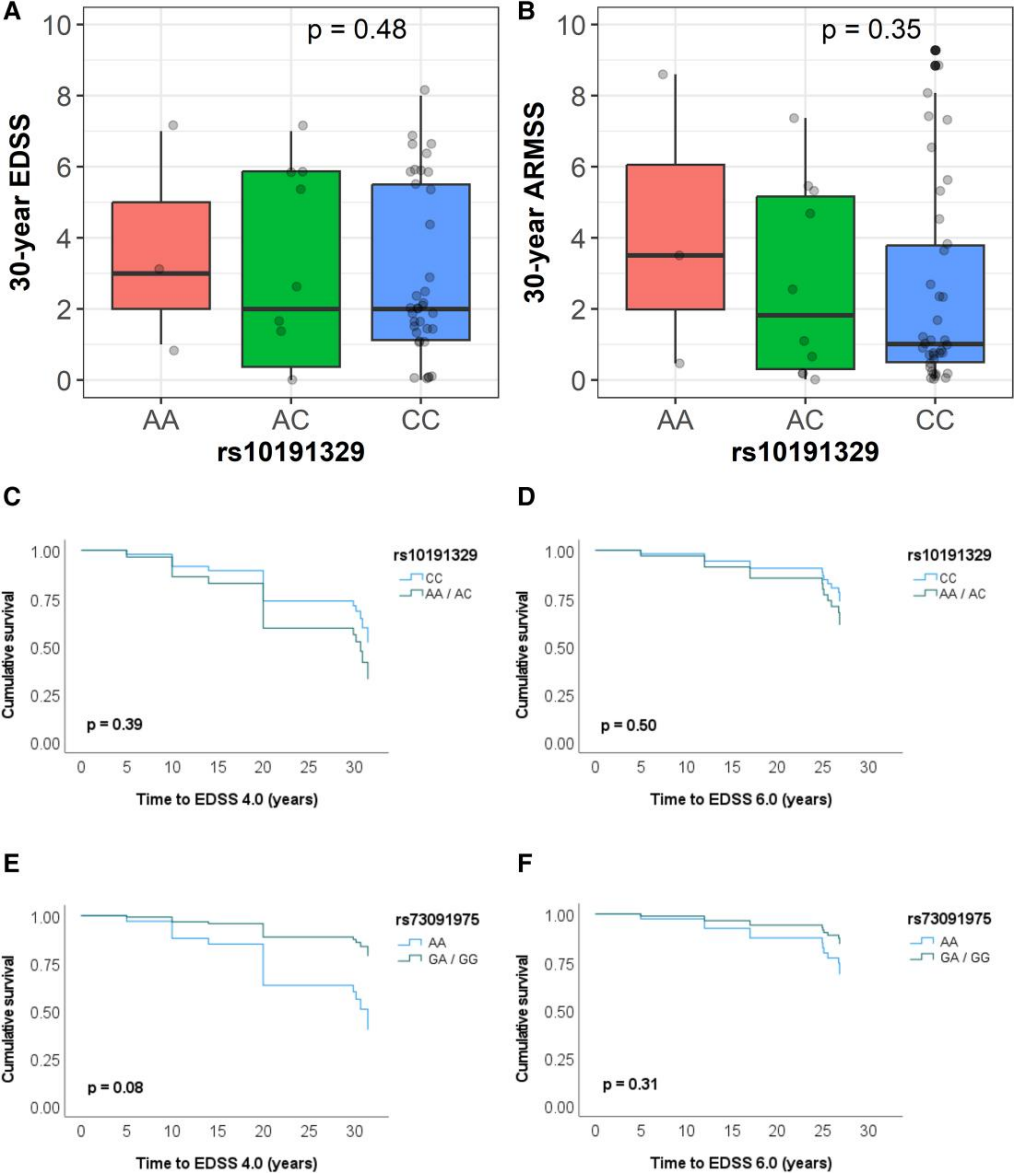
**Associations of rs10191329^A^ dosage with 30-year disability measures and rs10191329^A^ and rs73091975^G^ risk allele carriage with time to disability outcomes. (A** and **B)** Thirty-year disability scores for each participant by rs10191329 genotype are shown by the data points. No significant differences were observed in linear regression models assessing associations of rs10191329^A^ dosages with **(A)** EDSS or **(B)** ARMSS at 30-year follow-up, *n*  *=* 51 for both. Survival analysis curves (assuming dominant genetic models) using Cox proportional hazards models **(C–F)** found no associations of **(C)** rs10191329 with time to EDSS 4.0 (maximum walking ability 500 m without aid) and **(D)** rs10191329 with time to EDSS 6.0 (requiring a walking aid), *n*  *=* 39 for both. **(E)** A trend towards association was seen for rs73091975 with time to EDSS 4.0 but not with **(F)** rs73091975 and time to EDSS 6.0, *n*  *=* 40 for both. All models shown were adjusted for age, sex, DMT use and smoking history.

There were 3 homozygous carriers of the rs10191329^A^ allele and 10 heterozygous carriers within the 51 participants. No association was found between rs10191329^A^ dosage and EDSS [β-est = +0.4, 95% confidence interval (CI): (−0.8, 1.7), *P* = 0.48] or ARMSS [β-est = +0.7, 95% CI: (−0.7, 2.0), *P* = 0.35] at 30 years (Fig. 1A and B ). Although rs10191329^A^ dosage was associated with higher EDSS [β-est = +0.9, 95% CI: (0.08, 1.7), *P* = 0.03] at 14 years in whole group analyses, this was not replicated in the multiple sclerosis–only group [β-est = +0.9, 95% CI: (−0.1, 1.9), *P* = 0.08], and no associations were seen with either EDSS or ARMSS at other cross-sectional timepoints (Table 2). Using a dominant model instead of an additive model showed similar results, as did collider bias testing (Supplementary Tables 4–6).

**Table 2:** Associations of rs10191329^A^ with disease severity measures by follow-up

Follow-up	Whole group (*n* = 51)				Multiple sclerosis only (*n* = 39)			
	EDSS		ARMSS		EDSS		ARMSS	
	β (SE)	*P*-value	β (SE)	*P*-value	β (SE)	*P*-value	β (SE)	*P*-value
0	−0.2 (0.3)	0.59	−0.1 (0.6)	0.92	−0.1 (0.4)	0.72	0.0 (0.7)	0.97
5	0.2 (0.3)	0.47	0.2 (0.6)	0.68	−0.0 (0.4)	0.99	−0.6 (0.7)	0.41
10	0.3 (0.4)	0.39	0.3 (0.6)	0.59	0.1 (0.4)	0.81	−0.6 (0.7)	0.44
14	0.9 (0.4)	0.03*	1.2 (0.6)	0.07	0.9 (0.5)	0.08	0.6 (0.8)	0.44
20	0.9 (0.5)	0.08	0.9 (0.6)	0.14	0.8 (0.6)	0.19	0.8 (0.7)	0.25
30	0.4 (0.6)	0.48	0.7 (0.7)	0.35	0.2 (0.8)	0.78	0.5 (0.9)	0.59

Beta-coefficients (β) with standard error (SE) and *P*-values obtained from linear regression models assessing associations of rs10191329^A^ dosage with EDSS and ARMSS at each timepoint were adjusted for age, sex, DMT use and smoking history. Bonferroni correction for number of timepoints and outcomes (0.05/12) = *P*  *<* 4.2 × 10^−3^.

^*^ *P* < 0.05.

These findings were consistent with survival analyses of people who developed multiple sclerosis (*n*  *=* 39), which showed no association of rs10191329 with either time to EDSS 4.0 (*P* = 0.39) or time to EDSS 6.0 (*P* = 0.50; Fig. 1C and D; Supplementary Table 7).

rs10191329^A^ was not associated with cortical lesions, GMF or risk of developing SPMS at 30 years in the whole group, or in the multiple sclerosis–only subgroup. No association was seen with important early inflammatory predictors of SPMS status in this cohort (presence of baseline infratentorial lesion or deep white matter lesions at 1 year)^7^ or white matter lesion volume at 30 years (Supplementary Table 8).

### rs73091975^G^ is associated with age-related multiple sclerosis severity score at 14 years

No MSBase variants were associated with 30-year disease severity outcomes. The rs73091975^G^ allele was associated with ARMSS at 14-year follow-up in the whole group [β-est = −2.1, 95% CI: (−3.7, −0.5), *P* = 0.012] and in those with multiple sclerosis [β-est = −2.7, 95% CI: (−4.4, −0.9), *P* = 4 × 10^−3^]. Consistent with this effect, an association of rs73091975^G^ was seen with 30-year GMF in the multiple sclerosis group [β-est = +0.9%, 95% CI: (0.05, 1.8), *P* = 0.039; Table 3]. Survival analyses of rs73091975^G^ dosage found no association with time to EDSS 6.0 [hazard ratio = 0.45, 95% CI: (0.10–2.11), *P* = 0.31] or time to EDSS 4.0 [hazard ratio = 0.26, 95% CI: (0.06–1.18), *P* = 0.08; Fig. 1E and F; Supplementary Table 7]. The only other association was of rs7758683^T^ with 30-year white matter lesion volume [β-est = −7.2 ml, 95% CI: (−14.0, −0.49), *P* = 0.036].

**Table 3:** Associations of multiple sclerosis base variants with cross-sectional outcome measures

Variant (minor allele)	Call rate	MAF	Outcome measure	β (SE)	*P*-value
rs7758683 (T)	0.96	0.23	White matter lesion volume at 30 years, ml	−7.2 (3.4)	0.036*
rs73091975 (G)	0.96	0.15	ARMSS at 14 years in whole group	−2.1 (0.8)	0.012*
			ARMSS at 14 years in multiple sclerosis–only subgroup	−2.7 (0.8)	3.9 × 10^−3^**
			GMF at 30 years in multiple sclerosis–only subgroup, %	0.9 (0.4)	0.039*

Beta-coefficients (β) and *P*-values obtained from linear regression models assessing associations of variant allele dosage with outcome measures were adjusted for age, sex, DMT use and smoking history.

MAF, minor allele frequency.

^*^ *P* < 0.05.

^**^Bonferroni correction for number of timepoints and outcomes (0.05/12) = *P*  *<* 4.2 × 10^−3^.

## Discussion

In this prospectively acquired and deeply phenotyped cohort of people with CIS followed for 30 years, we found no association of rs10191329 with multiple sclerosis disease severity, pathological findings associated with disease progression (cortical lesions or brain atrophy) or with long-term risk of developing SPMS at 30 years. There was also no association of the imputed MSBase variants with 30-year disease severity, although we did replicate the association of rs73091975 with lower ARMSS in multiple sclerosis at 14 years.

Although genetic associations with 30-year disability outcomes were not replicated, the deep phenotyping (both clinical and radiological), alongside extensive and homogenous 30-year follow-up, provide unique insights into genetic associations that might inform future studies. The overall disease duration of participants, 30.9 years versus 18.2 years (IMSGC) and 18.1 years (MSBase) or other replication analyses (14 years^5^ and earlier^2^), helps distinguish people who will or will not develop substantial and progressive disability. By 30 years, the cohort assessed here showed clear clinical and MRI differences, and the mean time to develop SPMS was 19.6 years after first symptom onset. Studies of a shorter duration are likely to classify people who will develop SPMS as having RRMS and may fail to capture significant disability accrued later in the disease course due to disease progression rather than relapses. Further, it is possible for genetic factors to influence different elements of multiple sclerosis pathology, and in our earlier study, we found evidence that factors influencing relapses and white matter lesion accrual (HLA-DRB1*1501) were not associated with 30-year outcomes.^10^

This cohort was recruited before multiple sclerosis DMTs became routinely available and was largely untreated [32/39 (82%) of those who developed multiple sclerosis], compared with <10% in the IMGSC study,^1^ the MSBase register (median 79.7% time on DMT),^4^ 80.6% on DMT at follow-up in the study by Gasperi *et al.*^2^ and similar to Kreft *et al*.^5^ (73.1% untreated). This allows a clearer assessment of direct genetic effects on the natural history of disease progression, while associations in other studies may be dampened by effects from DMT reducing long-term disability. This may explain the greater effect sizes (albeit with wide error margins) observed here for both rs10191329 and rs73091975 in multiple sclerosis participants. For rs10191329, although not significant, the greatest effect sizes per rs10191329^A^ allele were seen between 14 and 20 years (0.6- to 0.8-point greater ARMSS) versus 0.071-point higher ARMSS in the IMSGC study (meta-analysis of discovery and replication cohorts, mean disease duration of 17.1 years). Similarly, the greatest effect for rs73091975 was seen at 14 years (2.7-point lower ARMSS) compared with the MSBase study (0.68-point lower ARMSS at median disease duration of 18.1 years).

The decline in effect sizes at 30 years suggests potential for non-linear genetic associations with multiple sclerosis severity. While this may reflect the non-linear trajectory of disability progression,^19^ effects observed from individual genetic variants may depend on the biological processes they affect, e.g. rs10191329 being implicated in neuroinflammation rather than CNS resilience may explain earlier maximal effects on disease severity.^20^ As multiple sclerosis severity is a complex trait, governed by both polygenicity and environmental (non-genetic) factors,^21^ some genetic effects may also be contingent on environmental conditions (gene–environment interactions) unaccounted for in genome-wide associations studies (GWAS).^22^ For example, genes involved in regulatory pathways for immune responses may only impact disability if exposed to a DMT acting on such pathways. Cohorts with greater exposure to this DMT may reveal genetic associations with disability, but those with lower exposure to this DMT would not.

The main limitation of this study is the small cohort size which, despite the observation of greater effect sizes, limits statistical power to replicate large-scale GWAS findings, particularly in low-frequency variants. Nonetheless, the present cohort has proven sufficiently large to power the detection of associations with other variants previously^10^ and here replicated the association of rs73091975 with ARMSS at the 14-year timepoint. However, other associations reported in this study would not withstand correction for multiple comparisons and require further validation, and negative findings do not preclude associations that may be detectable in a larger cohort. Furthermore, while survival analyses assumed dominant genetic models to maximize statistical power, additive models may be more biologically appropriate. Selection bias may also have negated potential effects in this cohort; as genotyping was only performed at the 30-year follow-up, the most severely affected individuals (due to earlier death or severe disability precluding attendance) have been partially selected out. This is reflected in the lower disability scores in the multiple sclerosis group (mean EDSS 3.4 and mean ARMSS 3.0) compared with the IMSGC (mean EDSS 3.5 and mean ARMSS 4.2) and MSBase (median longitudinal ARMSS 4.13) cohorts, although our greater follow-up duration likely increases the proportion of irreversible disability acquired during disease progression.

Additionally, genetic associations may have been limited by population admixture as ethnicity was not formally recorded in our study. While our cohort recruited in 1980’s London is likely to be predominantly of European ancestry, both MSBase and IMSGC cohorts were exclusively so and replication attempts in other ethnic groups were unsuccessful, albeit underpowered.^1^ Although ethnicity may influence MS severity, it remains unclear whether this is mediated by direct effects of ancestral genetic variation on disease severity or associated with disparities in health care and socio-economic factors.^23,24^

## Conclusion

This study found no association of rs10191329 with 30-year disease severity, grey matter pathology (cortical lesions or brain atrophy) or SPMS disease course. MSBase variants were similarly not associated with 30-year disease severity, although the association of rs73091975 with ARMSS in multiple sclerosis was replicated at 14 years despite the relatively small cohort size. These findings further highlight the complexity in replicating genetic associations with long-term multiple sclerosis disease severity and suggest future studies may need to account for genetic effects varying non-linearly with disease duration.

## Supplementary Material

fcae443_Supplementary_Data

## Data Availability

Anonymized data not published in the article may be shared upon reasonable request from a qualified investigator.
