# Supplementary material for: Evaluating multiple sclerosis severity loci 30 years after a clinically isolated syndrome
Source: Brain Commun. 2024 Dec 5;6(6):fcae443. doi: 10.1093/braincomms/fcae443 (PMC11635286; doi:10.1093/braincomms/fcae443)
Supplement: fcae443_Supplementary_Data [file fcae443_supplementary_data.pdf]

Supplementary Table 1 – Clinical and radiological characteristics by 30-year clinical diagnosis in participants genotyped for rs10191329

		All genotyped participants	Participants tested for rs10191329	Diagnosis at 30 years		
				CIS	RRMS	SPMS
**Number**		61	51	12	24	15
**Age (years)**		60.9 ± 6.5	60.9 ± 6.5	60.6 ± 6.8	60.6 ± 6.4	61.9 ± 6.7
**Rs10191329***	*AA*	NA	3 (6%)	1	2	0
	AC		10 (20%)	1	6	3
	CC		38 (75%)	10	16	12
**Female**		41 (67%)	34 (67%)	7 (58%)	16 (67%)	11 (73%)
**Age at Onset (years)**		30.2 ± 6.4	30.2 ± 6.7	29.5 ± 7.5	29.9 ± 6.8	31.6 ± 6.2
**Disease Duration (years)**		30.8 ± 0.9	30.9 ± 0.9	30.8 ± 0.9	31.0 ± 0.9	30.8 ± 0.9
**CIS Type**	*Optic Neuritis*	31 (51%)	25 (49%)	7 (58%)	10 (42%)	8 (53%)
	Spinal cord	21 (34%)	17 (33%)	4 (33%)	8 (33%)	5 (33%)
	Brainstem	9 (15%)	9 (15%)	1 (8%)	6 (25%)	2 (13%)
**Baseline EDSS**[a]	*Mean ± SD*	2.6 ± 1.3	2.6 ± 1.3	3.3 ± 1.2	2.3 ± 0.9	2.5 ± 1.7
	Median (IQR)	3.0 (2.0-3.125)	3.0 (2.0-3.25)	3.0 (3.0-3.5)	2.0 (2.0-3.0)	3.0 (1.0-3.5)
**Time CIS to RRMS (years)**		5.8 ± 6.0	5.4 ± 5.4	NA	6.4 ± 6.3	3.8 ± 2.9
**Time CIS to SPMS (years)**		19.6 ± 5.5	19.6 ± 5.5	NA	NA	19.6 ± 5.5
**EDSS at 30 years**	*Mean ± SD*	2.7 ± 2.4	2.9 ± 2.5	1.1 ± 1.1	1.9 ± 1.6	6.2 ± 0.8
	Median (IQR)	2.0 (1.0-5.5)	2.0 (1.0-6.0)	0.75 (0.0–2.0)	1.5 (1.0-2.0)	6.0 (6.0-6.5)
**DMT usage**	*Yes*	9	7	0	2	5
	No	52	44	12	22	10
**Baseline WM lesion volume (ml)**		1.17 ± 2.37	1.31 ± 2.54	0.13 ± 0.20	0.86 ± 0.81	2.46 ± 3.94
**WM lesion volume at 30 years (ml)**		16.49 ± 14.23	17.70 ± 14.51	5.86 ± 9.41	17.85 ± 12.26	26.95 ± 14.64
**Cortical lesions at 30 years (*n*)**		0.7 ± 1.3	0.7 ± 1.3	0.0 ± 0.0	0.1 ± 0.2	2.2 ± 1.5
**GMF at 30 years (%)**		43.4 ± 1.3	43.4 ± 1.4	43.8 ± 1.2	43.7 ± 1.0	42.5 ± 1.7

CIS – clinically isolated syndrome, RRMS – relapsing remitting multiple sclerosis, SPMS – secondary progressive multiple sclerosis, EDSS – expanded disease severity scale, IQR – interquartile range, DMT – disease modifying therapy, WM – white matter, GMF – grey matter fraction

Mean ± standard deviations unless stated otherwise

* Minor allele frequency= 0.15

[a] Baseline EDSS was recorded during initial CIS presentation.

Supplementary Table 2 – Genotype distribution of successfully imputed MSBase variants

**Variant (minor allele)**	*Call rate*	*MAF*	*Genotype Distribution*					
**rs7758683 (T)**	0.96	0.23	TT	2 (4%)	TG	19 (37%)	GG	30 (59%)
**rs73091975 (G)**	0.96	0.15	GG	0 (0%)	GA	15 (29%)	AA	36 (71%)
**rs7289446 (G)**	0.96	0.36	GG	5 (10%)	GA	25 (49%)	AA	21 (41%)
**rs1207401 (A)**	0.98	0.35	AA	5 (10%)	AG	25 (48%)	GG	22 (42%)
**rs56194930 (G)**	0.96	0.08	GG	0 (0%)	GC	8 (16%)	CC	43 (84%)
**rs295254 (G)**	1.00	0.48	GG	12 (23%)	GA	25 (47%)	AA	16 (30%)
**rs11057374 (G)**	1.00	0.39	GG	8 (15%)	GA	24 (45%)	AA	21 (40%)

MAF – minor allele frequency

Supplementary Table 3 – Clinical and radiological characteristics by 30-year clinical diagnosis in participants genotyped for rs73091975

		All genotyped participants	Participants tested for rs73091975	Diagnosis at 30 years		
				CIS	RRMS	SPMS
**Number**		61	51	11	26	14
**Age (years)**		60.9 ± 6.5	61.1 ± 6.7	61.0 ± 7.6	60.9 ± 6.6	61.5 ± 6.7
**Rs73091975**	*GG*	NA	0 (0%)	-	-	-
	GA		15 (29%)	3	10	2
	AA		36 (71%)	8	16	12
**Female**		41 (67%)	36 (71%)	6 (55%)	18 (69%)	10 (71%)
**Age at Onset (years)**		30.2 ± 6.4	30.2 ± 6.5	30.3 ± 7.4	29.9 ± 6.6	30.8 ± 6.2
**Disease Duration (years)**		30.8 ± 0.9	30.8 ± 0.9	30.7 ± 1.0	31.0 ± 0.9	30.7 ± 0.9
**CIS Type**	*Optic Neuritis*	31 (51%)	25 (49%)	7 (64%)	11 (42%)	8 (73%)
	Spinal cord	21 (34%)	17 (33%)	4 (36%)	9 (35%)	4 (36%)
	Brainstem	9 (15%)	9 (15%)	0	6 (23%)	2 (18%)
**Baseline EDSS**[a]	*Mean ± SD*	2.6 ± 1.3	2.6 ± 1.3	3.2 ± 1.3	2.3 ± 1.0	2.6 ± 1.7
	Median (IQR)	3.0 (2.0-3.125)	3.0 (2.0-3.25)	3.0 (3.0-3.5)	2.0 (2.0-3.0)	3.0 (1.0-3.5)
**Time CIS to RRMS (years)**		5.8 ± 6.0	5.8 ± 5.8	NA	6.7 ± 6.7	3.9 ± 3.0
**Time CIS to SPMS (years)**		19.6 ± 5.5	19.4 ± 5.7	NA	NA	19.4 ± 5.7
**EDSS at 30 years**	*Mean ± SD*	2.7 ± 2.4	2.9 ± 2.5	1.2 ± 1.2	1.8 ± 1.5	6.2 ± 0.8
	Median (IQR)	2.0 (1.0-5.5)	2.0 (1.0-5.5)	1.5 (0.0–2.0)	1.5 (1.0-2.0)	6.0 (5.875-6.625)
**DMT usage**	*Yes*	9	6	0	2	4
	No	52	45	11	24	10
**Baseline WM lesion volume (ml)**		1.17 ± 2.37	1.38 ± 2.61	0.15 ± 0.21	0.86 ± 0.81	2.66 ± 4.07
**WM lesion volume at 30 years (ml)**		16.49 ± 14.23	17.81 ± 14.44	5.83 ± 9.87	17.53 ± 11.80	27.71 ± 14.89
**Cortical lesions at 30 years (*n*)**		0.7 ± 1.3	0.7 ± 1.3	0.0 ± 0.0	0.0 ± 0.2	2.3 ± 1.5
**GMF at 30 years (%)**		43.4 ± 1.3	43.4 ± 1.4	43.9 ± 1.2	43.7 ± 0.9	42.5 ± 1.7

CIS – clinically isolated syndrome, RRMS – relapsing remitting multiple sclerosis, SPMS – secondary progressive multiple sclerosis, EDSS – expanded disease severity scale, IQR – interquartile range, DMT – disease modifying therapy, WM – white matter, GMF – grey matter fraction

Mean ± standard deviations unless stated otherwise

[a]Baseline EDSS was recorded during initial CIS presentation.

Supplementary Figure 1 – Proportional hazards assumptions testing for Cox regression models

A - rs10191329 and Time to EDSS 4.0

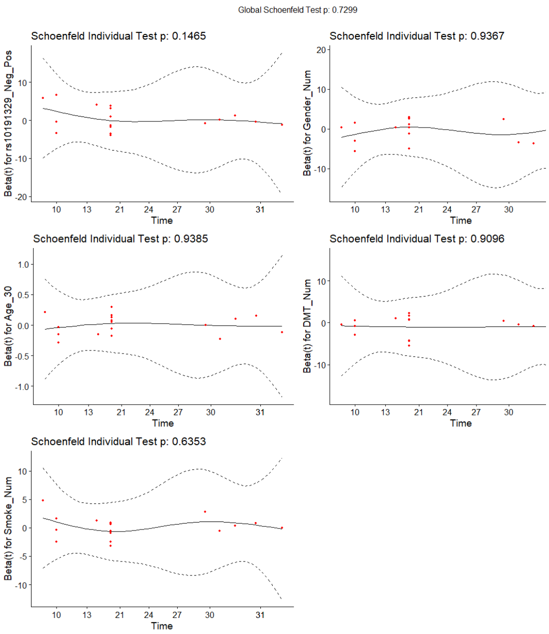

B - rs10191329 and Time to EDSS 6.0

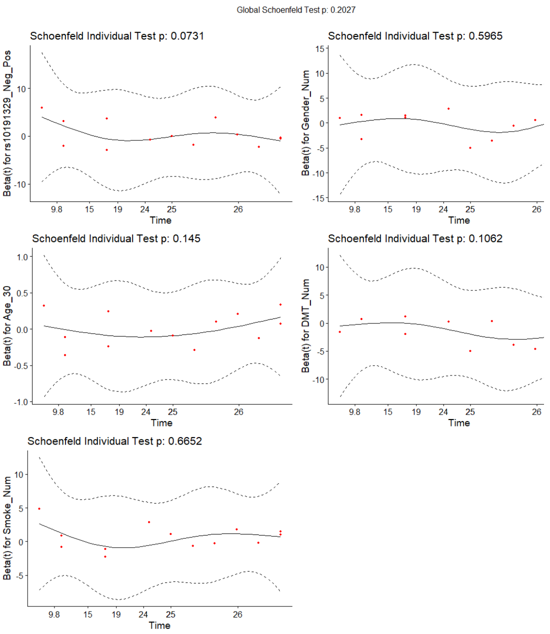

C - rs73091975 and Time to EDSS 4.0

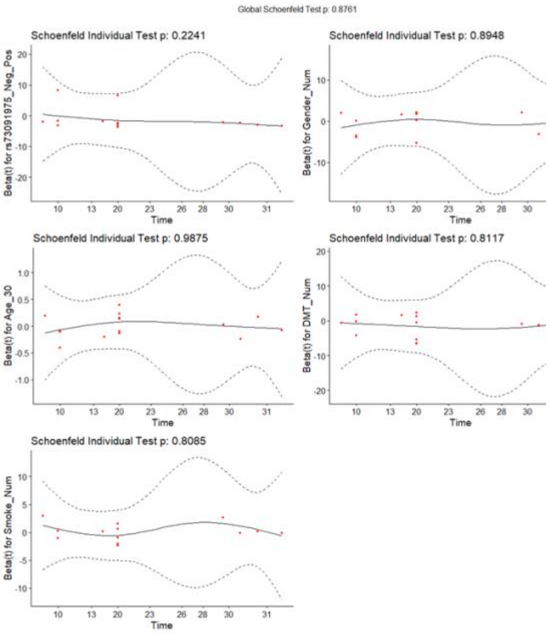

D - rs73091975 and Time to EDSS 6.0

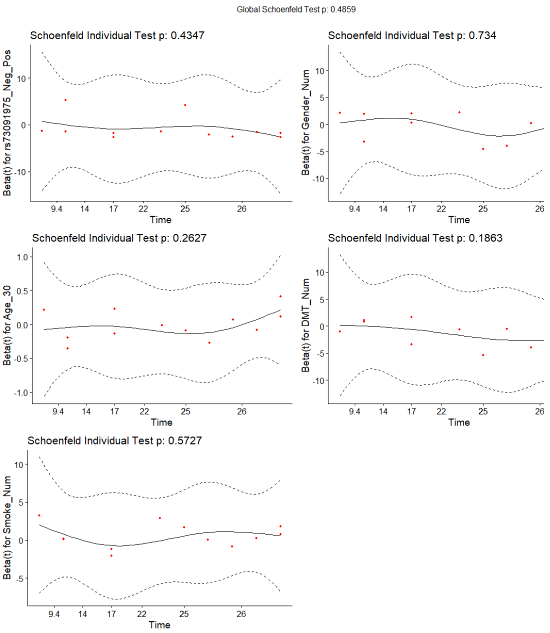

EDSS – Expanded Disability Status Scale

(**A-D**) Graphs of the scaled Schoenfeld residuals against time for the four Cox proportional hazards models assessing the association of rs10191329 and rs73091975 with time to disability outcomes. Each datapoint represents the scaled Schoenfeld residual for each individual experiencing an event. The proportional hazards assumption for Cox regression models was tested and supported by assessment of the Schoenfeld residuals with time for each model (global test) and for each covariate within the model (individual test), which showed no significant relationship between residuals and time ($p > 0.05$).

Supplementary Table 4 – Associations of rs10191329 with disease severity measures by follow-up assuming a dominant model

Follow-up	Whole group (*n=51)*				MS only *(n=39)*			
	EDSS		ARMSS		EDSS		ARMSS	
	β (SE)	p-value	β (SE)	p-value	β (SE)	p-value	β (SE)	p-value
0	-0.2 (0.4)	0.67	-0.1 (0.7)	0.93	-0.1 (0.5)	0.86	0.1 (0.9)	0.91
5	0.4 (0.4)	0.30	0.5 (0.8)	0.51	0.3 (0.5)	0.52	-0.0 (0.9)	0.96
10	0.4 (0.5)	0.41	0.3 (0.9)	0.75	0.2 (0.5)	0.65	-0.9 (1.0)	0.38
14	0.9 (0.5)	0.10	1.5 (0.9)	0.12	0.9 (0.6)	0.15	0.6 (1.1)	0.58
20	0.9 (0.7)	0.20	0.9 (0.8)	0.29	0.8 (0.8)	0.34	0.8 (0.9)	0.38
30	0.4 (0.8)	0.66	0.6 (0.9)	0.51	0.1 (1.0)	0.91	0.4 (1.1)	0.73

MS – multiple sclerosis; EDSS – Expanded Disability Status Scale; ARMSS – age-related MS severity,
Beta-coefficients *(*β) with standard error (SE) and p-values obtained from linear regression models assessing associations of rs10191329 assuming a dominant model (AA/AC vs CC) with EDSS and ARMSS at each timepoint adjusted for age, sex, disease-modifying therapy use and smoking history.

Supplementary Table 5 – Associations of rs10191329[A] with disease severity measures by follow-up including collider bias testing

Follow-up	Whole group (*n=51*)								MS only *(n=39)*							
	EDSS[a]		EDSS[b]		ARMSS[a]		ARMSS[b]		EDSS[a]		EDSS[b]		ARMSS[a]		ARMSS[b]	
	β (SE)	p-value	β (SE)	p-value	β (SE)	p-value	β (SE)	p-value	β (SE)	p-value	β (SE)	p-value	β (SE)	p-value	β (SE)	p-value
0	-0.2 (0.3)	0.59	-0.2 (0.3)	0.57	-0.1 (0.6)	0.92	-0.1 90.6)	0.89	-0.1 (0.4)	0.72	-0.2 (0.4)	0.66	0.0 (0.7)	0.97	-0.1 (0.7)	0.93
5	0.2 (0.3)	0.47	0.2 (0.3)	0.47	0.2 (0.6)	0.68	0.3 (0.6)	0.64	-0.0 (0.4)	0.99	-0.0 (0.4)	0.99	-0.6 (0.7)	0.41	-0.5 (0.7)	0.51
10	0.3 (0.4)	0.39	0.3 (0.4)	0.45	0.3 (0.6)	0.59	0.3 (0.6)	0.61	0.1 (0.4)	0.81	0.0 (0.4)	0.91	-0.6 (0.7)	0.44	-0.6 (0.7)	0.43
14	0.9 (0.4)	**0.03***	0.9 (0.4)	**0.04***	1.2 (0.6)	0.07	1.1 (0.6)	0.10	0.9 (0.5)	0.08	0.9 (0.5)	0.09	0.6 (0.8)	0.44	0.5 (0.8)	0.52
20	0.9 (0.5)	0.08	0.8 (0.5)	0.12	0.9 (0.6)	0.14	0.8 (0.6)	0.21	0.8 (0.6)	0.19	0.7 (0.6)	0.25	0.8 (0.7)	0.25	0.7 (0.7)	0.34
30	0.4 (0.6)	0.48	0.3 (0.7)	0.62	0.7 (0.7)	0.35	0.5 (0.7)	0.46	0.2 (0.8)	0.78	0.0 (0.8)	0.96	0.5 (0.9)	0.59	0.3 (0.9)	0.74

MS – multiple sclerosis; EDSS – Expanded Disability Status Scale; ARMSS – age-related MS severity,

Beta-coefficients (β) with standard error (SE) and p-values obtained from linear regression models assessing associations of rs10191329[A] dosage with EDSS and ARMSS at each timepoint

[a] - adjusted for age, sex, disease-modifying therapy use and smoking history.

[b] – collider bias tested by adjusting for age, sex, and smoking history but not disease-modifying therapy use.

***** $p < 0.05$**

NB: Bonferroni correction for number of timepoints and outcomes (0.05/12) = $p < 4.2 \times 10^{-3}$

Supplementary Table 6 – Associations of rs73091975[G] with disease severity measures by follow-up including collider bias testing

Follow-up	Whole group (*n=51*)								MS only *(n=40)*							
	EDSS[a]		EDSS[b]		ARMSS[a]		ARMSS[b]		EDSS[a]		EDSS[b]		ARMSS[a]		ARMSS[b]	
	β (SE)	p-value	β (SE)	p-value	β (SE)	p-value	β (SE)	p-value	β (SE)	p-value	β (SE)	p-value	β (SE)	p-value	β (SE)	p-value
0	0.3 (0.4)	0.94	0.0 (0.4)	0.92	-0.3 (0.7)	0.69	-0.3 (0.7)	0.72	-0.3 (0.5)	0.53	-0.3 (0.5)	0.56	-0.6 (0.8)	0.46	-0.6 (0.8)	0.50
5	-0.3 (0.4)	0.37	-0.3 (0.4)	0.36	-0.9 (0.8)	0.26	-0.9 (0.7)	0.24	-0.5 (0.4)	0.24	-0.5 (0.4)	0.23	-0.9 (0.8)	0.27	-1.0 (0.8)	0.24
10	-0.0 (0.4)	0.97	-0.0 (0.4)	0.99	-0.7 (0.8)	0.40	-0.6 (0.8)	0.40	-0.3 (0.5)	0.61	-0.2 (0.5)	0.61	-0.9 (0.8)	0.30	-0.9 (0.8)	0.29
14	-0.5 (0.5)	0.31	-0.5 (0.5)	0.33	-2.1 (0.8)	**0.01***	-2.1 (0.8)	**0.01***	-0.9 (0.6)	0.17	-0.9 (0.6)	0.17	-2.7 (0.8)	**3.9×10^{-3} ***	-2.7 (0.9)	**4.5×10^{-3} ***
20	-0.6 (0.6)	0.32	-0.6 (0.7)	0.35	-0.6 (0.8)	0.42	-0.6 (0.8)	0.47	-1.1 (0.7)	0.15	-1.1 (0.7)	0.16	-1.0 (0.9)	0.26	-1.0 (0.9)	0.30
30	-1.1 (0.7)	0.13	-1.1 (0.8)	0.16	-1.0 (0.8)	0.25	-0.9 (0.9)	0.29	-1.7 (0.9)	0.05	-1.7 (0.9)	0.06	-1.4 (1.0)	0.17	-1.4 (1.0)	0.19

MS – multiple sclerosis; EDSS – Expanded Disability Status Scale; ARMSS – age-related MS severity,

Beta-coefficients (β) with standard error (SE) and p-values obtained from linear regression models assessing associations of rs10191329[A] dosage with EDSS and ARMSS at each timepoint

[a] - adjusted for age, sex, disease-modifying therapy use and smoking history.

[b] – collider bias tested by adjusting for age, sex, and smoking history but not disease-modifying therapy use.

***** $p < 0.05$**

NB: Bonferroni correction for number of timepoints and outcomes (0.05/12) = $p < 4.2 \times 10^{-3}$

Supplementary Table 7 – Associations from survival analyses of rs10191329 and rs73091975 with time to disability milestones

Variant	Hazard of reaching disability milestone							
	EDSS 4.0[a]		EDSS 4.0[b]		EDSS 6.0[a]		EDSS 6.0[b]	
	HR (95% CI)	p-value	HR (95% CI)	p-value	HR (95% CI)	p-value	HR (95% CI)	p-value
**rs10191329** ***n = 39***	1.70 (0.50-5.72)	0.39	1.56 (0.48-5.04)	0.46	1.59 (0.42–6.01)	0.50	1.31 (0.38-4.56)	0.67
**rs73091975** ***n = 40***	0.26 (0.06-1.18)	0.08	0.29 (0.06-1.33)	0.11	0.45 (0.10-2.11)	0.31	0.50 (0.11-2.39)	0.39

EDSS – Expanded Disease Severity Scale, HR - hazard ratio, CI – confidence interval
Hazard ratios obtained from Cox proportional hazards models assessing association of genetic variant (assuming dominant genetic models) with time to disability milestone in participants who developed multiple sclerosis
[a] - adjusted for age, sex, disease-modifying therapy use and smoking history.
[b] – collider bias tested by adjusting for age, sex, and smoking history but not disease-modifying therapy use.

Supplementary Table 8 – Associations of rs10191329[A] with radiological measures at 30 years

Outcome measure	Whole group *(n=51)*		MS only *(n=39)*	
	β-est (95% CI)	*p*-value	β-est (95% CI)	*p*-value
Early predictors of 30-year SPMS status				
Baseline infratentorial lesion count, ≥ 1 vs 0 *(odds ratio)*	1.07 (0.22 to 5.25)	0.93	1.04 (0.19 to 5.68)	0.68
1-year deep white matter lesion count, ≥ 1 vs 0 *(odds ratio)*	0.69 (0.23 to 2.05)	0.50	0.30 (0.06 to 1.51)	0.14
Radiological outcomes at 30-year follow up				
Cortical lesions, *n*	0.04 (-0.65 to 0.74)	0.91	-0.08 (-1.03 to 0.87)	0.86
Grey matter fraction, %	-0.05 (-0.65 to 0.54)	0.86	-0.06 (-0.87 to 0.75)	0.88
White matter lesion volume, *ml*	-0.19 (-7.47 to 7.10)	0.96	-1.60 (-10.47 to 7.28)	0.72
Clinical outcomes at 30-year follow up				
Progression to SPMS, *(odds ratio)*	0.56 (0.13 to 2.36)	0.43	0.43 (0.09 to 1.96)	0.27

MS – multiple sclerosis; SPMS – secondary progressive multiple sclerosis, CI – confidence interval
Beta-coefficients (β) and *p*-values obtained from linear and logistic regression models assessing associations of rs10191329[A] dosage with outcome measures adjusted for age, sex, disease-modifying therapy use and smoking history.
